# A cross-sectional survey of the prevalence of environmental tobacco smoke preventive care provision by child health services in Australia

**DOI:** 10.1186/1471-2458-11-324

**Published:** 2011-05-17

**Authors:** Todd R Heard, Justine B Daly, Jennifer A Bowman, Megan AG Freund, John H Wiggers

**Affiliations:** 1 Hunter New England Population Health, New South Wales (NSW) Department of Health, Australia, (Longworth Avenue), Wallsend, NSW (2287), Australia; 2 Faculty of Health, The University of Newcastle, (University Drive), Callaghan, NSW (2308), Australia; 3 3 Faculty of Science and IT, The University of Newcastle, (University Drive), Callaghan, NSW (2308), Australia; 4 Hunter Medical Research Institute, (Lookout Road), New Lambton Heights, NSW (2305), Australia

**Keywords:** environmental tobacco smoke, paediatric health, preventative care, smoking, health facilities

## Abstract

**Background:**

Despite the need for a reduction in levels of childhood exposure to environmental tobacco smoke (ETS) being a recognised public health goal, the delivery of ETS preventive care in child health service settings remains a largely unstudied area. The purpose of this study was to determine the prevalence of ETS preventive care in child health services; differences in the provision of care by type of service; the prevalence of strategies to support such care; and the association between care support strategies and care provision.

**Method:**

One-hundred and fifty-one (83%) child health service managers within New South Wales, Australia completed a questionnaire in 2002 regarding the: assessment of parental smoking and child ETS exposure; the provision of parental smoking cessation and ETS-exposure reduction advice; and strategies used to support the provision of such care. Child health services were categorised based on their size and case-mix, and a chi-square analysis was performed to compare the prevalence of ETS risk assessment and ETS prevention advice between service types. Logistic regression analysis was used to examine associations between the existence of care support strategies and the provision of ETS risk assessment and ETS exposure prevention advice.

**Results:**

A significant proportion of services reported that they did not assess parental smoking status (26%), and reported that they did not assess the ETS exposure (78%) of any child. Forty four percent of services reported that they did not provide smoking cessation advice and 20% reported they did not provide ETS exposure prevention advice. Community based child and family health services reported a greater prevalence of ETS preventive care compared to other hospital based units. Less than half of the services reported having strategies to support the provision of ETS preventive care. The existence of such support strategies was associated with greater odds of care provision.

**Conclusions:**

The existence of major gaps in recommended ETS preventive care provision suggests a need for additional initiatives to increase such care delivery. The low prevalence of strategies that support such care delivery suggests a potential avenue to achieve this outcome.

## Background

Children are particularly vulnerable to the harmful effects of environmental tobacco smoke (ETS) because of their relatively underdeveloped immune and pulmonary systems, their small body size, and their higher rates of ventilation [1,2]. Exposure to ETS increases a child's risk of developing diseases of the respiratory system, otitis media and sudden infant death syndrome [1,3,4]. Further, child exposure to ETS is linked to increases in the frequency and severity of asthma symptoms, and both the onset of cardiovascular disease and permanent smoking behaviours in adulthood [1,3-5]. Hence, reducing child levels of exposure to ETS is a recognised public health goal [1,5].

Given that child exposure to ETS is most commonly associated with smoking by parents and other household member [6-8], interventions to reduce child levels of exposure have focused on encouraging parental smoking cessation and modifying parental smoking behaviours within the family home [9-11]. Such interventions have resulted in significant reductions in child levels of exposure to ETS [1,5,9]. Despite such reductions, a significant proportion of children continue to be exposed to ETS [1,4,6,12], and continued efforts to reduce childhood exposure to ETS are required [1,2,5,6].

Health facilities present an ideal opportunity to deliver ETS-prevention advice to parents [11]. First, they have the potential to reach up to 99% of parents of both well and sick children [13,14]. Second, research suggests that parents of well and sick children are receptive to ETS-prevention care delivered within health care settings [11]. Third, ETS-prevention interventions when delivered by health care professionals have been demonstrated to be efficacious [15-18]. Finally, the implementation of ETS-prevention care within health facilities is consistent with clinical best practice care delivery policies and guidelines [15,19,20].

Limited international evidence provides an insight into the level of ETS prevention care routinely provided in child health care settings. Such evidence is primarily United States based and addresses care provided by physicians only [14,21-23]. It suggests that health care professionals fail to routinely assess the ETS exposure of children, and to provide parents with advice on how to reduce child exposure to ETS [21,23,24]. Health care professionals cite a number of barriers to the provision of smoking cessation and other ETS-prevention related care, including a lack of time, skill and organisational support [11,21,23,25]. However, various strategies such as written policies, training, and reminder systems have been shown to be efficacious in facilitating clinical practice change generally, the provision of preventive care generally [26,27], and the provision of ETS preventive care specifically [21,28].

To date, research describing the provision of ETS-prevention care by heath care professionals, and the prevalence of strategies to support such care is limited. Given this, a study was undertaken to determine the: prevalence of ETS preventive care in child health services; the differences in care provision by type of child health services; the prevalence of strategies to support such care; and the association between care support strategies and care provision.

## Methods

### Study design and sample

A cross-sectional survey of public child health care services in New South Wales, Australia was undertaken in 2002. Eligible services were firstly, those units within hospitals that primarily cared for children aged 0-15 years of age, including all paediatric, postnatal and neo-natal units, and secondly, all community-based child and family health services. The eligible respondent for each health service was the Senior Nurse Manager within hospital settings, or the Senior Manager of child and family health services.

### Procedure

A self-report questionnaire was developed based on recommendations included in the United States' Clinical Guidelines for Treating Tobacco Use and Dependence [15], and on a previous Australian study that examined the prevalence of smoking cessation care provided to adult hospital patients [29]. A senior nurse unit manager within one health service pilot tested the survey structure and content, resulting in minor revisions before final distribution.

Pen and paper questionnaires were forwarded by the Chief Health Officer of New South Wales to all Health Services within the state. The Chief Executive of each service was asked to distribute the questionnaire to specified respondents within their organisation. Respondents were asked to complete the survey based on their knowledge of practices within their unit.

Health Services that did not return the surveys within three weeks received a telephone reminder prompt by the Chief Health Officer's staff. Ethics approval for human research was obtained from the University of Newcastle's Human Research Ethics Committee.

### Measures

#### Prevalence of ETS risk assessment

In the questionnaire respondents were asked to indicate if their unit assessed two forms of ETS risk: parental smoking status, and the ETS exposure of children ('yes', 'no' or 'unsure'). Those respondents, who indicated assessment was undertaken were asked to estimate the proportion of parents for whom each of form of assessment was routinely undertaken (1-25%, 26-50%, 51-75%, 76-99% or 100%).

#### Prevalence of ETS *e*xposure prevention advice

Respondents were asked whether their unit provided two forms of ETS exposure prevention advice to parents of children at risk: quit smoking advice and ETS reduction advice ('yes' 'no' or 'unsure'). Those respondents who indicated that advice was provided were asked to estimate the proportion of parents for whom such advice would be routinely provided (1-25%, 26-50%, 51-75%, 76-99% or 100%). Such respondents were also asked to indicate the type of advice offered (brief verbal advice, extended verbal advice, written materials, referral, and nicotine replacement therapy (NRT) ('yes' or 'no').

#### Prevalence of care support strategies

Respondents were asked if the provision ETS preventive care was supported by: documentation in the form of written policies; staff training; or organisational prompts such as computer based prompts and medical record stickers ('yes', 'no' or 'unsure').

#### Unit type

Respondents were asked to indicate the type of child health service (i.e. hospital unit or child and family health service) that they were reporting on.

### Analysis

#### Unit type

Hospitals were categorised into one of two groups based upon their size and case-mix. Group 1 hospitals included principal referral and major metropolitan and non-metropolitan hospitals treating between 10,000 and 25,000 patients per year. Group 2 hospitals included district and community hospitals treating between 2,000 and 10,000 patients per year. As a consequence, all units were clasified as either a Group 1 or Group 2 hospital, or a child and family health service.

#### Prevalence of ETS risk assessment and ETS exposure prevention advice

Responses regarding the estimated proportion of parents that routinely received either form of ETS risk assessment, and the proportion of parents that routinely received either form of ETS exposure prevention advice were collapsed into four categories: 'none' (0%), 'less than 75%' (1-75%), 'almost all' (76-99%) and 'all patients' (100%). Based on these categories, the overall prevalence of both ETS risk assessment and ETS exposure prevention advice were examined. Differences in the prevalence of each form of care between the types of services (Peer Group 1, Peer Group 2 and community-based Child and Family Health Service) were examined by chi-square analysis.

#### Prevalence of care support strategies

The prevalence of each type of care support strategy is reported as a proportion. Differences in the prevalence of such strategies between the types of services were examined by chi-square analysis.

#### Association between care support strategies and provision of ETS risk assessment, and ETS exposure prevention advice

Chi-square and logistic regression analyses were used to examine associations between the existence of care support strategies (independent variable) and each of the four forms of care (assessment of parental smoking, assessment of child ETS exposure, provision of quit smoking advice, and provision of ETS reduction advice) (dependent variables). For these analyses, responses regarding the proportion of patients receiving each form of care were collapsed into two groups, 0-75% of patients (some) and 76-100% of patients (most) [29]. Responses regarding the existence of each of the three care support strategies were dichotomised ('yes' or 'no'). Type of service was included as a controlling variable in the regression analyses. Variables with a p-value of 0.2 or less arising from the chi-square analysis were included in a multivariate logistic regression model. A backward stepwise multivariate logistic regression process was used, where the least statistically significant variable was removed from the regression model and the new model tested until all variables within the model were significant (*p *< 0.05). Evidence of confounding and possible interactions were investigated.

All analysis was undertaken using the statistical software package SAS 9.1.2 [30]. A finite population correction was applied to all analyses [31].

## Results

### Sample characteristics

Completed questionnaires were received from 153 of the 183 eligible units (83%). Response rates did not differ significantly between health service settings, or between hospital peer groups (range 81% to 84%). Seventy-two percent (n = 111) of respondents were from hospital units and 28% (n = 42) were from community-based child and family health services. Sixty one percent (n = 67) of hospital respondents were from Peer Group 1.

### Prevalence of ETS risk assessment

Twenty-six percent of all respondents indicated that their service did not assess the smoking status of any parents (see Table 1). Approximately half (52%) indicated that their facility routinely assessed parental smoking status of most parents (>75%). A greater proportion of respondents from community-based child and family health services (86%) reported assessing the smoking status of most parents (<75) compared to 33% of Peer Group 1 and 50% of Peer Group 2 respondents (*p *< 0.001).

**Table 1:** Assessment of parental smoking and child exposure to environmental tobacco smoke by type of health care unit

Assessment type	Patients receiving care	Peer Group 1		Peer Group 2		Child and family health unit		p value	Total (hospital + child family units)	
		n	%	n	%	n	%		n	%
**Parental smoking**	**0%**	31	45	7	17	2	4	<0.001	40	26
	**1-75%**	15	22	14	33	4	10		33	22
	**76-99%**	9	14	6	14	18	43		33	22
	**100%**	13	19	15	36	18	43		47	30
										
**Child ETS exposure**	**0%**	60	87	36	86	23	55	<0.001	119	78
	**1-75%**	6	9	3	7	4	10		13	9
	**76-99%**	1	1	1	2	7	17		9	6
	**100%**	2	3	2	5	8	19		12	8

Seventy-eight percent of all respondents, and 86%-87% of hospital units reported that their service did not assess the ETS exposure for any child. Fourteen percent indicated that their facility routinely assessed the ETS exposure for most children. A greater proportion of respondents from child and family health units reported assessing the ETS exposure of most children. Thirty-six percent of community-based child and family health respondents reported that they assessed the smoking status of most parents (>75%) compared to 4% of Peer Group 1 and 7% of Peer Group 2 respondents (*p *< 0.001).

### Prevalence of ETS exposure prevention advice

Nearly half of all respondents (44%) indicated that their service did not provide smoking cessation advice to any parents (see Table 2). A greater proportion of respondents from community-based child and family health services (71%) reported the provision of smoking cessation advice to at least some parents compared to 43% of Peer Group 1 respondents and 66% of Peer Group 2 respondents (*p *= 0.003).

**Table 2:** The provision of quit smoking and environmental tobacco smoke reduction advice by type of health unit

Advice type	Patients receiving care	Peer Group 1 units		Peer Group 2 units		Child and family health units		p value	Total (Hospital + Child family units)	
		n	%	n	%	n	%		n	%
Parental smoking Cessation*	**0%**	40	58	14	34	12	29	0.003	66	44
	**1-75%**	8	12	18	44	18	44		44	29
	**76-99%**	8	12	4	10	4	10		16	11
	**100%**	13	19	5	12	7	17		25	17
										
**Child ETS exposure reduction**^†^	**0%**	15	22	12	30	3	7	0.036	30	20
	**1-75%**	22	33	18	45	12	29		52	35
	**76-99%**	15	22	4	10	12	29		31	21
	**100%**	17	25	6	15	14	34		37	25

* data missing for two respondents

^† ^data missing for three respondents

Of those units that reported providing smoking cessation advice to parents (56%), 86% provided brief verbal advice, 75% provided written materials, 30% offered referrals, 22% provided extended verbal advice, 19% provided advice regarding NRT, and 13% provided NRT. Eighty percent of such units reported providing more than one type of quit smoking advice to parents.

Twenty percent of respondents indicated that their service did not provide ETS-reduction advice to any parents of children at risk, whilst approximately one half (46%) reported that such advice was provided to most parents. A greater proportion of respondents from community-based child and family health services reported providing ETS-reduction advice to most parents. Sixty-three percent of child and family health respondents reported that they provided ETS-reduction advice to most parents of children at risk compared to 47% of Peer Group 1 and 25% of Peer Group 2 respondents (*p *= 0.036).

Of those units that reported providing ETS reduction advice to parents (80%), 88% provided brief verbal advice, 65% provided written materials, 27% provided extended verbal advice, and 19% offered referrals. Seventy six percent of such units reported providing more than one type of ETS reduction advice to parents.

### Prevalence of care support strategies

As shown in Table 3, 22% of respondents reported that their service had a policy regarding the provision of smoking cessation care and 12% reported the existence of a policy regarding the provision of ETS exposure prevention. Forty-seven percent of services reported the provision of staff training regarding ETS preventive care and 20% reported the provision of prompts for the delivery of such care, respectively. Units did not differ in their report of the existence of either form of policy (*p *= 0.356 and *p *= 0.839 respectively) or the existence of prompts for the provision of ETS-prevention care (*p *= 0.781). Community-based child and family health unit respondents (68%) reported a higher prevalence of staff training in the provision of ETS-prevention care compared to Peer Group 1 (41%) and Peer Group 2 respondents (36%) (*p *= 0.007).

**Table 3:** Prevalence of care support strategies by type of health facility

	Peer Group 1 units		Peer Group 2 units		Child and family health units		*p *value	Total (Hospital + Child family health unit)	
	n	%	n	%	n	%		n	%
**Quit smoking policy**	8	24	10	29	6	15	0.356	24	22
**ETS prevention policy**	9	13	4	10	5	13	0.839	18	12
**Training**	28	41	15	36	27	68	0.007	70	47
**Prompts**	15	22	7	17	7	18	0.781	29	20

### Association between care support strategies and provision of ETS risk assessment

Independent of type of service, respondents who reported the existence of an ETS prevention policy had approximately twice the odds of assessing parental smoking status (odds ratio [OR] 2.31, confidence interval [CI] 1.40-3.81). Respondents who reported the existence of training regarding ETS prevention care had twice the odds of assessing parental smoking status (OR 2.14, CI 1.53-3.00) and over four times the odds of assessing child ETS exposure (OR 4.22, CI-1.71-10.42).

### Association between care support strategies and provision of ETS exposure advice

Independent of type of service, respondents reporting the existence of a quit smoking policy had nearly three times the odds of providing parental smoking cessation advice (OR 2.90, CI 1.04-8.12) (see Table 4). Respondents who reported the existence of an ETS prevention policy had over four times the odds of providing parental smoking advice (OR 4.41, CI 1.32-3.65) and nearly five times the odds of providing child ETS exposure advice (OR 4.91, CI 2.52-9.57). Respondents who reported the existence of prompts or reminders had nearly three times the odds of providing parental smoking advice (OR 2.75, CI 1.49-5.08) and over three times the odds of providing child ETS exposure advice (OR 2.34, CI 1.50-3.65).

**Table 4:** Association between provision of ETS-reduction interventions and unit type and support strategies

	Variable	Odds ratio	95% CI		***p***
			Lower	Upper	
**Assessment**					
Parental smoking					
	ETS prevention policy				
	Yes	2.31	1.40	3.81	0.001
	No	1.0			
					
	Training				
	Yes	2.14	1.53	3.00	<0.001
	No	1.0			
Child ETS exposure					
					
	Training				
	Yes	4.22	1.71	10.42	0.002
	No	1.0			
					
**Advice**					
Parental smoking					
					
	Quit smoking policy				
	Yes	2.90	1.04	8.12	0.043
	No	1.0			
					
	ETS prevention policy				
	Yes	4.41	1.32	14.74	0.016
	No	1.0			
					
	Prompts				
	Yes	2.75	1.49	5.08	0.001
	No	1.0			
					
Child ETS exposure					
	ETS prevention policy				
	Yes	4.91	2.52	9.57	<0.001
	No	1.0			
					
	Prompts				
	Yes	2.34	1.50	3.65	<0.001

The existence of an ETS prevention care policy significantly increased the odds of providing ETS exposure advice (OR 3.0 CI 1.3-6.6). No other associations were evident between service support strategies and the provision of such advice.

## Discussion

This study is the first that reports the prevalence of ETS preventive care routinely delivered by Australian public child health services. We found that a large proportion of such services failed to routinely assess ETS risks or provide ETS exposure prevention advice to parents of attending children. The results also suggest the limited existence of clinical practice strategies to support the provision of such care. The results indicate a considerable need and potential for additional clinical practice initiatives to realise the benefits of ETS prevention care for children.

The results of this study, despite methodological differences, are consistent with those of research in other countries that suggest that approximately half of child health services fail to assess the smoking status of the majority of parents [14,21,23,25]. For example, Winickoff et al surveyed 902 parents regarding the level of ETS prevention care provided by their family physician or paediatrician and found that 51% of parents reported being asked about the smoking status of household members [21]. Although the assessment of children's ETS exposure was found to occur at a lower level in the current study (14% of services routinely assessed ETS exposure), the observed level supports the findings of previous research that such assessment occurs at an inadequate level [21,25].

The results of the current study suggest that most services fail to routinely provide smoking cessation and ETS reduction advice to parents, which is consistent with previous research [14,21,23,25]. However, the current study's findings that 28% of services routinely provide smoking cessation advice to parents is lower than that reported previously [14,21,23,25]. In the study described above, Winickoff et al found 40% of parent smokers were advised to quit [21]. The current study's findings that 46% of services provided ETS exposure reduction advice to parents of children at risk is also consistent with previous studies [21,25].

The results of this study extend previous research by examining and demonstrating differences in the prevalence of ETS prevention care between types of services and clinicians [22,23]. The current study found that community-based child and family health services were more likely to assess both types of ETS risk. Similarly, community-based child and family health services, and Peer Group 2 hospital, more commonly provided parental smoking advice compared to Peer Group 1 hospitals, and community-based child and family health services were more likely to provide ETS reduction advice. Generally, the higher prevalence of care delivery in community-based child and family health services may be explained by their focus on 'well child' assessments and care, in comparison to the more acute clinical focus of hospitals [32]. The variability in care delivery between care settings suggests that a greater understanding of the differences in values, policies, systems and care delivery contexts between settings is required in order to facilitate the development of appropriately tailored interventions.

Evidence suggests that intervention strategies that are most effective in increasing ETS prevention care are system level strategies such as policies [33], reminder strategies or multi-component approaches inclusive of reminders and education [28]. The evidence suggests that multi-strategic approaches are more likely to be successful as they address the known multi-dimensional nature of barriers to the provision of such care [34]. Multi-strategic intervention approaches are also supported by review evidence regarding the provision of smoking cessation care [15,35], and changing clinical practice more generally [36,37].

The findings of this study support such previous research, by suggesting that where clinical practice is supported by a written policy, training and care prompts, clinicians are more likely to assess children's ETS risk and provide ETS prevention care. Despite this positive finding, it is evident that the majority of child health services do not incorporate support strategies within their organisations. Training regarding ETS prevention care was the most often reported support mechanism (47%) followed by ETS care prompts (20%), the existence of a policy outlining the provision of smoking cessation care (22%), and policy outlining ETS reduction care (12%). Such outcomes are similar to those reported in the adult inpatient setting [29].

The observed low levels of organisational support can be argued to have contributed to the finding that the majority of children attending child health services are not being assessed for ETS risk, or provided ETS prevention care. As these findings occur in the context of international and national guidelines that mandate the provision of ETS prevention interventions [15,38], the findings suggest additional initiatives to enhance the provision of these forms of care are required.

The findings of this study should be considered in the context of a number of its methodological characteristics. First, the study relied on the use of an indirect estimate of care prevalence by utilising self-report data of senior unit managers. Results obtained from self-report studies can be confounded as respondents commonly over-report the routine provision of assessment and advice [39]. If this was the case, the observed low frequency of reported provision of ETS prevention care represents an over estimation of actual practice, strengthening the conclusion that further care support initiatives are required. Given the limitations of both direct and proxy measures of clinical practice, further research should consider the use of a triangulation of multiple data collection methods [40]. Secondly, in some instances there were small frequencies in some cells for analysis and therefore conclusions regarding predictors of care should be considered exploratory. Finally, care should be taken when generalising these finding to child health services outside of Australia because of the unknown impact of differing child health service structures

## Conclusions

There is a clear need to further enhance the provision of ETS prevention care in child health services. Considering the documented efficacy of care support strategies such as reminders, education and policy, the further dissemination of ETS preventive care guidelines should incorporate such strategies.

## Competing interests

The authors declare that they have no competing interests.

## Authors' contributions

TH contributed to: conception and design of the study, acquisition of data, analysis and interpretation of the data, drafting of the manuscript, provided statistical expertise, read and approved the final manuscript.

JD contributed to: conception and design of the study, acquisition of data, analysis and interpretation of the data, drafting of the manuscript, provided supervision, read and approved the final manuscript.

JB contributed to: conception and design of the study, analysis and interpretation of the data, drafting of the manuscript, revision of the manuscript for important intellectual content and provided supervision, read and approved the final manuscript.

MF contributed to: conception and design of the study, analysis and interpretation of the data, drafting of the manuscript, revision of the manuscript for important intellectual content and provided supervision, read and approved the final manuscript.

JW contributed to: conception and design of the study, acquisition of data, analysis and interpretation of the data, drafting of the manuscript, revision of the manuscript for important intellectual content, involved in obtaining funding, providing supervision, read and approved the final manuscript.

## Financial disclosure

This study was undertaken with the support of Hunter New England Population Health and infrastructure support from the Hunter Medical Research Institute.

The sources of funding for the study, for each author, are as follows:

TH: Hunter New England Population Health, New South Wales (NSW) Department of Health, Australia; Faculty of Psychology, The University of Newcastle, NSW, Australia; Hunter Medical Research Institute, NSW Australia.

JD: Hunter New England Population Health, New South Wales (NSW) Department of Health, Australia; Faculty of Health, The University of Newcastle, NSW, Australia; Hunter Medical Research Institute, NSW Australia.

JB: Faculty of Health, The University of Newcastle, NSW, Australia; Hunter Medical Research Institute, NSW Australia.

MF: Hunter New England Population Health, New South Wales (NSW) Department of Health, Australia; the Faculty of Health, The University of Newcastle, NSW, Australia; Newcastle Institute of Public Health, NSW Australia; Hunter Medical Research Institute, NSW Australia.

JW: Hunter New England Population Health, New South Wales (NSW) Department of Health, Australia; Faculty of Health, The University of Newcastle, NSW, Australia; Newcastle Institute of Public Health, NSW Australia; Hunter Medical Research Institute, NSW Australia.

## Pre-publication history

The pre-publication history for this paper can be accessed here:

http://www.biomedcentral.com/1471-2458/11/324/prepub
